# High-Dose Chemotherapy with Autologous Hematopoietic Stem Cell Transplantation in Relapsed or Refractory Primary CNS Lymphoma: A Retrospective Monocentric Analysis of Long-Term Outcome, Prognostic Factors, and Toxicity

**DOI:** 10.3390/cancers14092100

**Published:** 2022-04-23

**Authors:** Sabine Seidel, Verena Nilius-Eliliwi, Thomas Kowalski, Deepak Ben Vangala, Uwe Schlegel, Roland Schroers

**Affiliations:** 1 Department of Neurology, University Hospital Knappschaftskrankenhaus, Ruhr University Bochum, In der Schornau 23-25, D-44892 Bochum, Germany; thomas.kowalski@kk-bochum.de (T.K.); uwe.schlegel@kk-bochum.de (U.S.); 2 Department of Hematology and Oncology, University Hospital Knappschaftskrankenhaus, Ruhr University Bochum, In der Schornau 23-25, D-44892 Bochum, Germany; verena.nilius-eliliwi@ruhr-uni-bochum.de (V.N.-E.); deepak.vangala@ruhr-uni-bochum.de (D.B.V.)

**Keywords:** primary central nervous system lymphoma, relapse, r/r PCNSL, high dose chemotherapy with autologous stem cell transplantation, carmustin/BCNU, thiotepa

## Abstract

**Simple Summary:**

No standard treatment is defined for relapsed or refractory (r/r) primary CNS lymphoma (PCNSL). However, high-dose chemotherapy with autologous stem cell transplantation (HCT-ASCT), an efficient first line treatment in younger PCNSL patients, is commonly applied in relapsed or refractory disease, if tolerable. Here, we retrospectively analyzed 59 patients with r/r PCNSL focusing specifically on differences in long-term outcome and toxicity in patients <65 years (*n* = 33) and ≥65 years (*n* = 26) of age.

**Abstract:**

High-dose chemotherapy with autologous stem cell transplantation (HCT-ASCT) is reportedly an effective treatment strategy in relapsed or refractory primary CNS lymphoma (r/r PCNSL); however, only selected patients are eligible for this treatment. We retrospectively analyzed outcome, prognostic factors, and toxicity in 59 patients with r/r PCNSL planned to receive HCT-ASCT at our institution between January 2005 and December 2021 (*n* = 33 < 65 years; *n* = 26 ≥ 65 years). Median follow-up was 65 months (95% CI 21–109). Median age was 63 years (range 29–76), median Karnofsky performance score (KPS) was 80 (range 30–100). In the entire cohort of 59 patients, median overall survival (OS) was 14 months (95% CI 0–37). In 50/59 (84.7%) patients who completed HCT-ASCT, median progression free survival (PFS) was 12 months (95% CI 3–21) and median OS 30 months (95% CI 0–87). 1-year, 2-year, and 5-year OS rates of 61.2%, 52.3% and 47.1%, respectively, were observed. Six patients (10.2%) died related to treatment (1 during induction treatment, 5 post HCT-ASCT). Age was not prognostic. On univariate analysis, KPS ≥ 80 (*p* = 0.019) and complete or partial remission before HCT-ASCT (*p* = 0.026) were positive prognosticators of OS; on multivariate analysis, KPS (*p* = 0.043) and male gender (*p* = 0.039) had an impact on OS. The 5-year OS rate in patients with progressive or stable disease after induction treatment was 32.7%. In summary, HCT-ASCT was effective and feasible in this cohort of r/r PCNSL patients. Clinical state, remission status before HCT-ASCT, and gender influenced survival, whereas age did not influence outcome in this study.

## 1. Introduction

High-dose chemotherapy with autologous stem cell transplantation (HCT-ASCT) is an intensive treatment strategy used in various types of hematological malignancies, solid tumors, and autoimmune disorders [1]. While formerly HCT-ASCT was almost exclusively applied to patients <65 years, it has been increasingly used also in selected patients up to 75 years in the last decades [2].

In newly diagnosed primary central nervous system lymphoma (PCNSL), HCT-ASCT is a commonly applied and effective treatment strategy for younger patients (≤60 years [3], ≤65 years [4], ≤70 years [5,6]) in the first line setting and has shown promising results in selected patients ≥65 years [7,8]. In relapsed or refractory PCNSL (r/r PCNSL), HCT-ASCT can also result in durable lymphoma remissions. Two prospective trials on HCT-ASCT in r/r PCNSL that included only younger patients reported on 2-year overall survival (OS) rates of 45% [9] and 56% [10], respectively. In a retrospective study on 79 patients (of which six were older than 65 years) with PCNSL or primary ocular lymphoma, who completed HCT-ASCT, a 2-year OS rate of 68% was reported [11]. The rates of treatment-related deaths ranged between 7.0% and 10.3% [9,10,11].

In this study, we retrospectively analyzed survival, prognostic factors, and toxicity in 59 consecutive patients with r/r PCNSL referred to our hospital and considered eligible for HCT-ASCT by more liberal criteria, including patients up to the age of 76 years. This analysis specifically focuses on differences in outcome and toxicity in patients <65 years (*n* = 33) and patients ≥65 (*n* = 26).

## 2. Patients and Methods

All consecutive patients with r/r PCNSL for whom HCT-ASCT was planned as salvage treatment at our hospital between January 2005 and December 2021 were included in this analysis, irrespective of their clinical performance status. All patients were HIV negative. Patients were included in cases of radiologically confirmed relapse of PCNSL, progression of PCNSL during or after completion of first line treatment or partial response only after completion of initial treatment.

Staging at relapse comprised whole body FDG-PET or CT of neck, chest, and abdomen, bone marrow biopsy, ophthalmological examination, and cerebrospinal fluid (CSF) diagnostics. Baseline examinations prior to HCT-ASCT included evaluation of comorbidities (using the Charlson Comorbiditiy Index [12]) of renal, bone marrow and pulmonary function, echocardiography, and dental examination.

We included all patients planned for HCT-ASCT with relapsed or refractory PCNSL referred to our hospital. Therapy with HD-MTX during first line treatment was not mandatory for inclusion in this analysis. HCT-ASCT was applied as second- or third-line treatment in this cohort. In the majority of patients, high dose chemotherapy comprised rituximab, thiotepa, and carmustin (R-TT-BCNU) [4,10]. One patient treated in 2006 was treated without rituximab as reported in the first publication of the protocol [13]. One patient received a busulfan-based HCT protocol in 2005. If more than 1 year had passed between last high-dose methotrexate (HD-MTX)-based chemotherapy and relapse of PCNSL, HD-MTX retreatment (four cycles of HDMTX 4000 mg/m^2^ in combination with rituximab 375 mg/m^2^) was planned prior to induction. Induction chemoimmunotherapy consisted of two courses of rituximab 375 mg/m^2^ (day 1), cytarabine 3000 mg/m^2^ (days 2 and 3), and thiotepa 40 mg/m^2^ (day 3). Between the first and second induction cycle, autologous peripheral blood stem cells were collected following granulocyte colony-stimulating factor (G-CSF) stimulation. Conditioning high-dose chemoimmunotherapy consisted of rituximab 375 mg/m^2^ (day −7), BCNU 400 mg/m^2^ (day −6) and thiotepa 2 × 5 mg/kg (days −5 and −4) followed by reinfusion of autologous hematopoietic cells on day 0 and G-CSF starting on day +4 (see also Supplementary Figure S1).

Responses were evaluated according to the International Primary CNS Lymphoma Collaborative Group (IPCG) criteria [14]. Toxicity was graded using Common Terminology Criteria for Adverse Events (CTCAE, version 5.0). Patients were included in a follow-up program at our hospital including regular clinical and ophthalmological examination as well as cerebral magnet resonance imaging (cMRI) according to International Primary CNS Lymphoma Collaborative Group (IPCG) guidelines [14]. Examinations were scheduled quarterly for 2 years after completion of therapy, every 6 months for 3 more years, and annually afterwards, comparable to recommendations for follow-up after completion of first line treatment [15]. CSF examinations were only conducted if leptomeningeal relapse was suspected.

Patient characteristics, clinical, laboratory, and imaging data were collected by chart review. The censoring date was 31 January 2022. The Ethics Committee of the University of Bochum, Faculty of Medicine approved the study (21-7212-BR). One patient included in this analysis had participated in a previously published prospective trial on HCT-ASCT in r/r PCNSL [10].

Median follow-up was calculated using the inverse Kaplan–Meier method. OS was calculated from the first day of salvage treatment to death of any cause or last date of follow-up. Progression free survival (PFS) was defined as the time from first day of salvage treatment to progression, death of any cause (if progression was not determined) or last date of follow-up. OS and PFS were estimated by the Kaplan–Meier method. To compare OS and PFS between groups, log-rank tests were used. Multivariate analysis was performed using the Cox proportional hazard regression model. The level of significance was 0.05 (two-sided). For the reported variables, complete data was available for all patients. Variables for multivariate analysis were chosen based on previously published series on HCT-ASCT in r/r PCNSL [7,9,10,11] with a supplementary analysis of the Charlson comorbiditiy score as a potential prognosticator. Analyses were conducted using SPSS (version 23).

## 3. Results

### 3.1. Patient Characteristics

**Figure 1:**
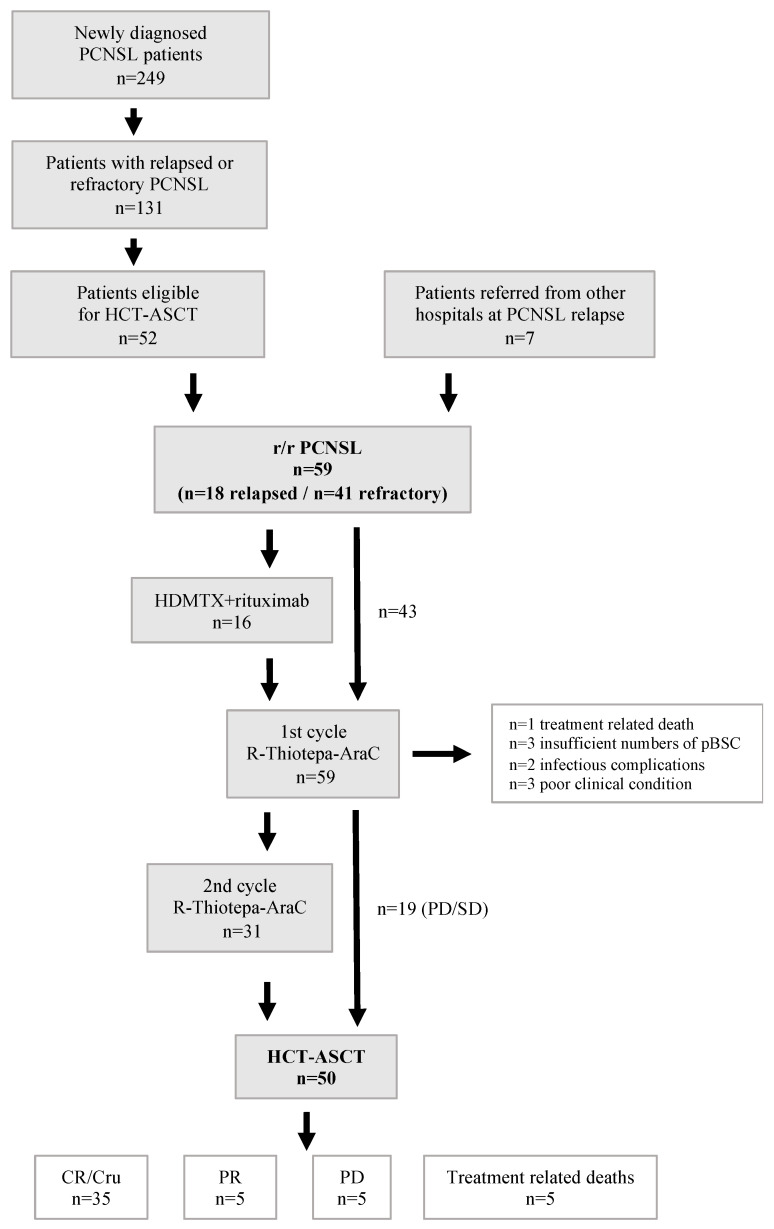
Flow chart of treatments and responses of patients with r/r PCSNL. Abbreviations: CR/Cru—complete remission/complete remission unconfirmed, HCT-ASCT—high dose chemotherapy with autologous stem cell transplantation, HDMTX—high dose methotrexate, r/r—relapsed/refractory, PCNSL—primary CNS lymphoma, pBSC—peripheral blood stem cells, R-Thiotepa-AraC—rituximab, thiotepa, cytarabine.

Between January 2005 and December 2021, a total of 249 patients with initial diagnosis of PCNSL have been treated at our hospital. Of these, 131 patients had refractory disease at first line treatment or experienced relapse after first line treatment with HD-MTX-based polychemotherapy and 52 were eligible for and started induction treatment prior to HCT-ASCT. Seven patients who had received first line treatment at a different institution and HCT-ASCT as second line treatment at our hospital were also included in this analysis. Thus, a total of 59 patients was analyzed in this study (*n* = 41 relapsed, *n* = 18 refractory; Figure 1).

Median follow-up was 65 months (95% CI 21–109 months). Four patients (6.8%) were lost to follow-up. Median age at HCT-ASCT was 63 years (range 29–76 years), and median Karnofsky performance score (KPS) was 80 (range 30–100). In all patients, histopathological diagnosis was diffuse large B-cell lymphoma. All patients received HCT-ASCT at cerebral relapse, no patient had ocular relapse nor systemic lymphoma. Two patients were on immunosuppressive medication (*n* = 1 methotrexate for chronic polyarthritis, *n* = 1 continuous corticosteroids for polymyalgia rheumatica).

First line treatment had been HD-MTX-based polychemotherapy according to a modified “Bonn” protocol [16] in 50 patients (*n* = 7 including intrathecal liposomal cytarabine, *n* = 33 including intracerebroventricular therapy via Ommaya reservoir; Supplementary Table S1, 5 patients had been treated according to the original “Bonn” protocol [17], 1 patient had received 6 cycles of HD-MTX monotherapy, 2 patients had received 6 cycles of HD-MTX and ifosfamide (in 1 patient followed by whole brain radiation [WBRT]), and 1 patient had been treated with 6 cycles of doxorubicin, cyclophosphamide, vincristine, and prednisone (CHOP). This patient received four cycles of rituximab and HD-MTX at relapse before induction treatment with R-Thiotepa-AraC.

Responses to first line treatments had been complete remissions (CR) or complete remissions unconfirmed (CRu) in 41 (69.5%) patients, partial remissions (PR) in 2 (3.4%) patients, and progressive disease (PD) in 16 (27.1%) patients, respectively.

Median time from initial diagnosis to PCNSL recurrence in relapsed patients was 16.5 months (range 1–60 months). Median time from last day of prior treatment to relapse with subsequent HCT-ASCT was 13 months (range 2–68 months).

Four patients received HCT-ASCT as third line treatment; in these patients, second line treatments had been WBRT (*n* = 1), WBRT and HD-MTX (*n* = 1), rituximab and temozolomide (*n* = 1), and ocular radiation therapy with intraocular rituximab at ocular relapse (*n* = 1), respectively. Detailed patient characteristics are given in Table 1.

### 3.2. High-Dose Chemotherapy and Response

In 3 patients, insufficient numbers of peripheral blood stem cells were collected precluding HCT-ASCT. One of these patients received WBRT, and two patients were treated by palliative care. One treatment-related death due to sepsis was observed subsequent to induction chemotherapy. In 5 patients, treatment was stopped prematurely during or early after initial chemotherapy because of significant infectious complications (*n* = 2) or overall poor clinical condition (*n* = 3).

Overall, 50 patients (84.7%) of our cohort completed HCT-ASCT. Seventeen of these patients had received only 1 cycle of rituximab, cytarabine, and thiotepa before HCT-ASCT because of progression (*n* = 16) or stable disease (*n* = 1) after the first treatment cycle.

In 49 patients, high dose chemotherapy comprised rituximab, carmustin (BCNU), and thiotepa. One patient received a busulfan-based HCT-ASCT regimen (in 2005). A median of 5.2 × 10^6^ CD34+ peripheral blood stem cells/kg bodyweight were reinfused. The median time to neutrophil and platelet engraftment was 9 and 10 days, respectively.

Responses after completion of HCT-ASCT were complete remission (CR) or complete remission unconfirmed (CRu) in 35 of 50 patients (70.0%), PR in 5 patients (10.0%), and PD in 5 patients (10.0%) as determined by cMRI 2–3 months post HCT-ASCT. Treatment-related death associated to HCT-ASCT occurred in 5 (10%) patients.

Three of five patients with PR after HCT-ASCT were treated by best supportive care; in one patient, WBRT was administered, and in one patient, six cycles of temozolomide stabilized the PCNSL for 13 months. A summary of treatment courses and responses for all patients is displayed in Figure 1.

### 3.3. Survival Analyses and Prognostic Factors

**Figure 2:**
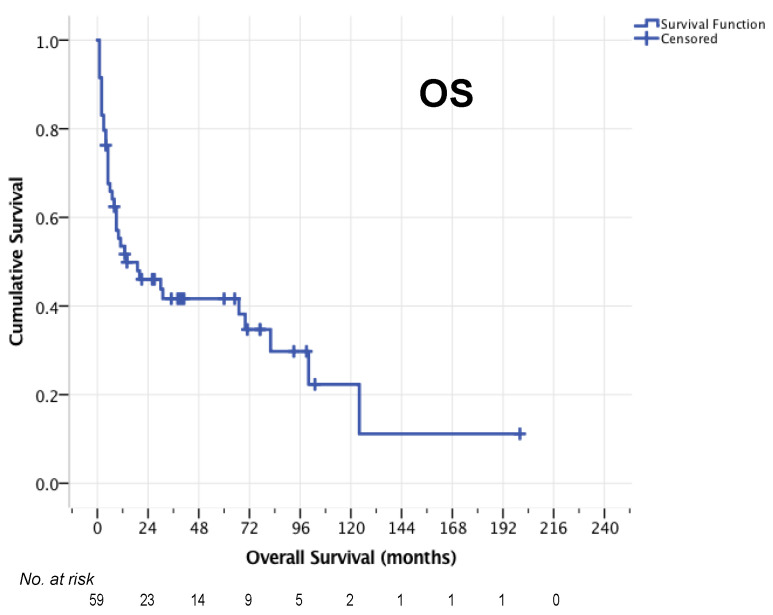
OS of the entire cohort (*n* = 59).

**Figure 3:**
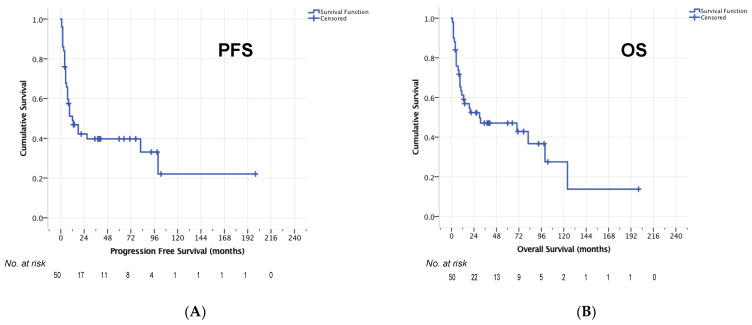
PFS (**A**) and OS (**B**) in 50 patients who completed HCT-ASCT.

Median OS after relapse for the entire cohort was 14 months (95% CI 0–37 months, Figure 2). The 1-year OS rate was 53.5%, 2-year OS rate 46.0% and 5-year OS-rate 41.6%. For 50/59 patients who completed HCT-ASCT, the median PFS was 12 months (95% CI 3–21, Figure 3a) and median OS was 30 months (95% CI 0–87 months, Figure 3b). Here, 1-year, 2-year, and 5-year OS rates of 61.2%, 52.3% and 47.1% were observed, respectively. The 1-year PFS rate was 49.0%, the 2-year PFS rate 42.2% and the 5-year PFS rate 39.7%.

Five patients died more than 5 years after relapse with planned HCT-ASCT. One of these patients had not received HCT-ASCT because of insufficient stem cell collection. This patient died of lymphoma relapse. Cause of death in the other patients were a myelodysplastic syndrome with secondary ALL (*n* = 1), severe encephalopathy in a patient who had received whole brain radiotherapy at third relapse (*n* = 1), severe cachexia mainly caused by malnutrition due to an alcohol addiction (*n* = 1) and unknown (*n* = 1).

Next, the following factors potentially influencing prognosis were evaluated for patients who completed HCT-ASCT (*n* = 50): age groups (≥65 vs. <65, ≥50 vs. <50, ≥70 vs. <70), relapsed vs. refractory PCNSL, responses to induction treatment (CR/PR vs SD/PD), general physical performance at relapse (KPS ≥ 80 vs. <80), PCNSL involvement of deep brain structures, gender, and comorbidity (Charlson score 0 vs. ≥1).

**Figure 4:**
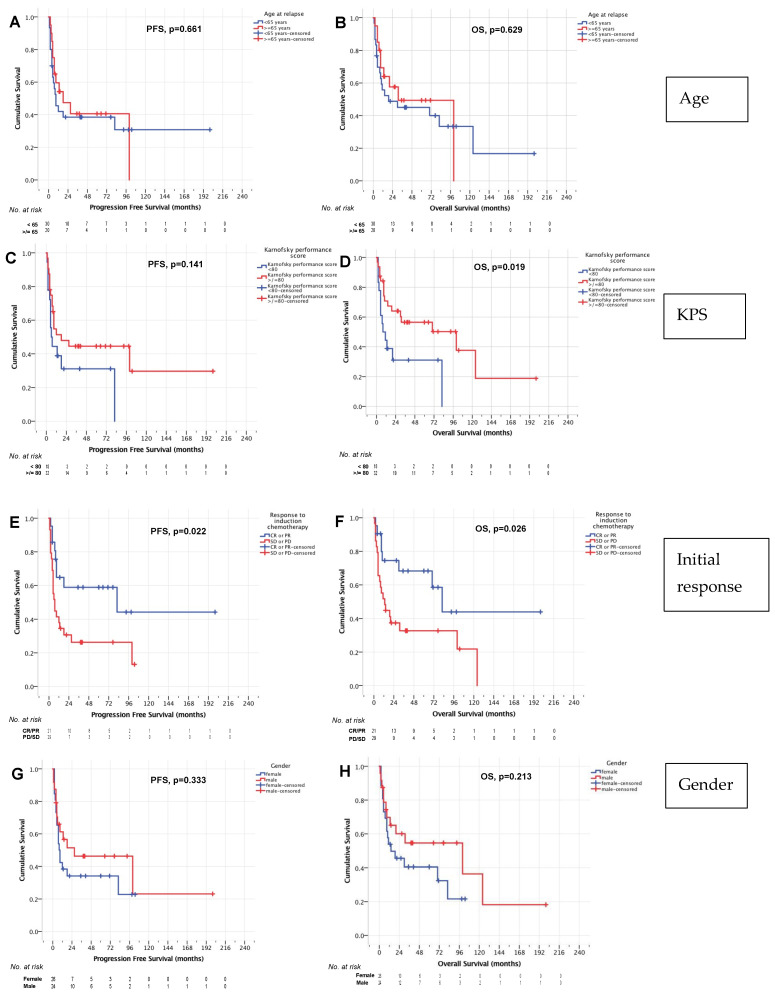
PFS and OS age (**A**,**B**), Karnofsky performance score (**C**,**D**), remission status before HCT-ASCT (**E**,**F**), and gender (**G**,**H**).

No significant influence of age on PFS or OS was found on univariate analysis. Median PFS was 18 months (95% CI 0–40 months) in patients ≥65 years and 9 months (95% CI 3–15 months, *p* = 0.661, Figure 4A) in patients <65 years. Median OS was 31 (95% CI 0–65 months) and 19 months (95% CI 0–87 months, *p* = 629, Figure 4B) in these age groups, respectively.

In addition, no significant impact of KPS at relapse on PFS was identified. Median PFS was 18 months (95% CI 2–34 months) in patients with a KPS ≥ 80 and 6 months for patients with a KPS < 80 (95% CI 2–10 months, *p* = 0.141, Figure 4C). However, there was a significant influence of KPS on OS in our cohort. In patients with a KPS ≥ 80 median OS was 100 months (95% CI 5–195 months), whereas patients with a KPS < 80 had a median OS of 8 months (95% CI 0–16 months, *p* = 0.019, Figure 4D).

Patients who responded to induction chemoimmunotherapy had a significantly better median PFS and OS as compared to patients with SD or PD after induction. For patients with CR or PR after induction, median PFS was 82 months (95% CI 0–212 months) and median OS was also 82 months (95% CI 56–108 months). For patients with SD or PD after induction treatments, median PFS was 7 months (95% 4–10 months, *p* = 0.022, Figure 4E) and median OS was 13 months (95% CI 4–22 months, *p* = 0.026, Figure 4F). Of note, despite failure of induction therapies in r/r PCNSL, the 5-year OS rates in those patients was 32.7%. In patients not responsive to induction treatment, no difference in OS (median OS 19 months [95% CI 3–35] and 11 months [95% CI 0–22 months], *p* = 0.724) was found for patients with PD after the first cycle (who proceeded directly to HCT-ASCT) or after the second cycle R-Thiotepa-AraC.

No significant difference in survival associated with gender was demonstrated in univariate analysis. Median PFS was 27 months (95% CI 0–82 months) in male patients and 8 months (95% CI 5–11 months, *p* = 0.333, Figure 4G) in female patients. Median OS was 100 months (95% CI 0–209 months) and 14 months (95% CI 0–36 months, *p* = 0.213, Figure 4H), respectively.

On multivariate analyses, remission (CR or PR) after induction chemotherapy was associated with longer PFS (HR 0.411, 95% CI 0.172–0.979, *p* = 0.045). Better clinical performance status (KPS ≥ 80, HR 2.406, 95% CI 1.026–5.643, *p* = 0.043) and male gender (HR 2.426, 1.046–5.627, *p* = 0.039) were positively prognostic for OS (Table 2).

### 3.4. Toxicity

Overall, 6 (10.2%) treatment-related deaths occurred in our cohort: one during induction treatment and five during HCT-ASCT (age range 47–71 years, three male and three female patients). Four of these six patients were younger than 65 years. One patient died for unknown reasons before cMRI control during rehabilitative treatment; the other five patients died due to sepsis. For three of these patients, no origin of infection could be detected; two were diagnosed with pneumonia.

As anticipated, all 50 patients who completed HCT-ASCT experienced grade 3 and 4 hematological toxicities. A median of 2 red-blood cell and 2 platelet units were transfused in the context of HCT-ASCT. Grade 3 and 4 non-hematological toxicities for all patients, separated by age groups, are summarized in Table 3.

Forty patients suffered from grade 3 and 4 infectious complications (*n* = 36 grade 3 and *n* = 4 grade 4). Infectious constellations were neutropenic fever with undetermined origin in most cases (*n* = 24), followed by urinary tract infection (*n* = 6), and bacterial pneumonia (*n* = 5). Colitis, catheter-associated infection (*n* = 3 respectively), cerebral aspergillosis, septic thrombophlebitis, and significant oral thrush (*n* = 1 respectively) occurred less often.

### 3.5. Salvage Treatment

Salvage treatments at first relapse after HCT-ASCT or in case of refractory disease were WBRT (*n* = 9), palliative treatment (*n* = 3), thiotepa and busulfan-based HCT-ASCT (*n* = 2), rituximab, HD-MTX, temozolomide (*n* = 1), HCT followed by allogeneic hematopoietic stem cell transplantation (*n* = 1), R-CHOP at systemic relapse (*n* = 1), systemic rituximab plus WBRT (*n* = 1), systemic rituximab with HD-MTX, temozolomide plus WBRT (*n* = 1), and unknown (*n* = 1). Salvage treatments at subsequent relapses and survival after HCT-ASCT are shown in Supplementary Table S2.

## 4. Discussion

In this retrospective study, we analyzed long-term outcome, prognostic factors, and toxicity of HCT-ASCT in a cohort of 59 patients with r/r PCNSL. Treatment options in the situation of r/r PCNSL are WBRT [18,19], HD-MTX rechallenge in case of late relapse [20,21], topotecan [22,23], temozolomide (+ rituximab) [24,25] or ifosfamide and etoposide in combination with cytarabine [26] or rituximab [27]. In addition, an increasing number of novel therapeutic agents and cellular immunotherapies is evaluated within clinical studies [28,29].

The most effective treatment strategy for r/r PCNSL currently available is HCT-ASCT; however, only a selected number of patients is eligible for intensive treatment. While in the past applied primarily in patients <65 years, HCT-ASCT is increasingly also used in elderly patients [2]. In another study, HCT-ASCT had been feasible and effective in a series on 52 r/r PCNSL patients ≥65 years who completed treatment (*n* = 15 first line, *n* = 37 second or third line treatment) [7]. Two patients (3.8%) within this series died related to treatment. The 2-year OS rate was 66% and 2-year PFS rate was 54% for patients who received HCT-ASCT as second or third line treatment.

Two prospective studies on HCT-ASCT in r/r PCNSL patients <66 years have been published to date. One reported a 2-year OS rate of 45%, 2-year-PFS rate of 43%, and a median OS of 18.3 months with a thiotepa, busulfan, and cyclophosphamide-based regimen [8]. In the other study, a 2-year OS rate of 56% and a 2-year PFS rate of 46% were reported [10] with the same thiotepa and BCNU-based protocol as administered in most of our patients. Median OS in this series had not been reached [10]. The rates of treatment-related deaths were comparable to our experiences (10%) with 7% [9] and 10% [10], respectively.

In the largest retrospective series on patients with r/r PCNSL (*n* = 68) or primary intraocular lymphoma (*n* = 11) completing HCT-ASCT (92% <65 years), the 2-year and 5-year OS rates were 68% and 51%, respectively [11]. In our cohort, the 2-year and 5-year survival rates for patients who completed HCT-ASCT were 50.2% and 47.5%. Our series included a higher number of patients not responsive to induction treatment (57.6% vs. 26.6%), possibly explaining the slightly inferior outcome. Clinical performance status, another possible reason for the difference in outcome, had not been reported in the retrospective series.

A distinctive feature of our study is the detailed analysis of differences in outcome and toxicity in patients <65 years (*n* = 34) versus patients ≥65 years (*n* = 26). While the cut-off for age varies between different studies on HCT-ASCT in PCNSL (≤60 years [3], ≤65 years [4], ≤70 years [5,6]), we chose the cut-off of 65 years based on recent clinical studies focused on elderly patients [7,8] prior to our analysis. After we gained knowledge that for a cut-off of 65 years, age is not prognostic in our cohort, we performed an additional analysis with cut-offs of 50 and 70 years; however, these analyses also did not reveal any influence of age on survival.

Age itself is an acknowledged independent prognostic factor in PCNSL [30,31] and had prognostic impact in a retrospective French series of 79 patients with r/r PCNSL [11]. In this series, median OS for patients >60 years was 22 months and median OS for patients <60 years was 86 months (*p* = 0.001). However, this study included only 6 patients >65 years. Importantly, in our series, no significant differences in OS or PFS according to age were observed, and neither was the rate of treatment-related death higher in elderly patients (four deaths in patients <65 years, two in patients ≥65 years). Nor did an analysis of toxic side effects CTCAE grade 3 or 4 divided by age groups show any relevant differences (Table 2). An explanation for this unexpected finding might be the thorough selection of patients concerning eligibility for HCT-ASCT, particularly in elderly patients. In our patient cohort, the comorbidity score did not differ in patients <65 years and ≥65 years (63.6% vs. 65.4% had a score of 0, Table 1), while for cancer patients in general, a higher rate of comorbidities in elderly patients would be expected [32].

While we did not find age to be prognostic in our patient population, physical status (KPS) at relapse was a significant factor of survival in our study in multivariate and univariate analyses. Patients with KPS ≥ 80 had a significantly longer median OS as compared to patients with KPS < 80. This observation indicates that clinical state before HCT-ASCT is a better prognosticator than age. Indeed, performance status (ECOG < 2 vs. ≥2) has previously been shown to be prognostic in a prospective study published by Soussain et al. [9].

Similar to the results of other studies in r/r PCNSL [7,9,10,11], the prognosis of patients who did not respond to induction treatment was less favorable compared to patients with PR or CR after induction treatment in our study. This highlights the need of better induction chemotherapy protocols to potentially improve the overall results following HCT-ASCT. In our cohort, patients with SD (*n* = 2) and PD (*n* = 27) after induction chemotherapy for r/r PCNSL were nevertheless treated with HCT-ASCT. Despite these unsatisfactory results after initial chemoimmunotherapy, the 5-year OS rate in those patients was 33.6% following HCT-ASCT. In contrast to current clinical practice in refractory/recurrent nodal aggressive lymphoma, this encouraging result strongly suggests HCT-ASCT with BCNU and thiotepa to be considered in patients with r/r PCNSL despite inadequate responses to initial chemoimmunotherapies.

Male gender was a significant positive prognostic factor in multivariate analysis in this series. While gender is not a commonly known prognostic factor in PCNSL, data from one large registry study had shown female gender to be a favorable prognostic factor in PCNSL [28]. However, chemotherapy is generally less well tolerated by women [33], which might partly explain the better outcome in men in the intensively treated patient population in our study.

The retrospective monocentric and retrospective design limits the generalizability of our results however; we analyzed consecutive “intention to treat” patients. Due to the monocentric design and the subsequent access to all patient charts and laboratory data, we were able to carry out a thorough analysis of prognostic factors and toxic side effects. Considering the limited patient numbers in our study, the statistical results, particularly of the subgroup analysis, will require further analysis in a larger study population.

In summary, the results of HCT-ASCT in r/r PCNSL highlight its curative potential and compares favorably to other salvage treatments. Studies on WBRT [18,19], topotecan [22,23] and temozolomide (with rituximab) [24,25] have reported median OS of 3.9–32 months; however, none of these therapeutic approaches was curative.

Future treatment perspectives for patients with relapse after HCT-ASCT might be a wide range of targeted therapies [34] and are certainly cellular immunotherapies, such as allogeneic stem cell transplantation [35] and chimeric antigen receptor (CAR) T-cell approaches [29].

The increasing role of HCT-ASCT as first line treatment for PCNSL in eligible patients may lead to a declining role of HCT-ASCT as salvage treatment. Nevertheless, this study data on a cohort of homogenously treated and long term-followed (median 66 months) patients with r/r PCNSL, including data on the effectivity and toxicity of HCT-ASCT, demonstrate encouraging long-term survival in a challenging clinical setting.

## 5. Conclusions

HCT-ASCT was effective and feasible in this cohort of r/r PCNSL patients irrespective of age. High KPS response to induction treatment and male gender were positive indicators of survival. HCT-ASCT resulted in a 5-year OS rate of 47.5%. In patients with resistance to induction chemoimmunotherapy, the 5-year OS was 33.6%. We believe our results to be supportive in the process of clinical decision making in the situation of r/r PCNSL.

## Figures and Tables

**Table 1:** Patient characteristics at relapse/progression (*n* = 59).

	All Patients (*n* = 59)	Patients < 65 Years (*n* = 33)	Patients ≥ 65 Years (*n* = 26)	*p*-Value ^a^
*Age*, *years*				
median (range)	63 (29–76)			
*KPS*				
Median (range)	80 (30–100)	80 (30–100)	80 (40–100)	0.642
≥80	36 (61.0%)	21 (63.6%)	15 (57.7%)	
<80	23 (39.0%)	12 (36.4%)	11 (42.3%)	
*Gender*				
male	31 (52.5%)	15 (45.5%)	16 (61.5%)	0.219
female	28 (47.5%)	18 (54.5%)	10 (38.5%)	
*Involvement of DBS*				
Yes	23 (39.0%)	14 (42.4%)	9 (34.6%)	0.541
No	36 (61.0%)	19 (57.6%)	17 (65.4%)	
*Number of lesions*				
unifocal	33 (55.9%)	15 (45.5%)	18 (69.2%)	0.124
multifocal	24 (40.7%)	16 (48.5%)	8 (30.8%)	
diffuse leptomeningeal	2 (3.4%)	2 (6.1%)	0	
*Lactate dehydrogenase level*				
normal	41 (69.5%)	23 (69.7%)	18 (69.2%)	0.674
elevated	14 (23.7%)	7 (21.2%)	7 (26.9%)	
not available	4 (6.8%)	3 (9.1%)	1 (3.8%)	
*CSF cytology at relpase*				
Positive	7 (11.9%)	4 (12.1%)	3 (11.5%)	0.376
Negative	26 (44.1%)	17 (51.5%)	9 (34.6%)	
not done	26 (44.1%)	12 (36.4%)	14 (53.8%)	
*Charlson Comorbidity Index*				
Score 0	38 (64.4%)	21 (63.6%)	17 (65.4%)	0.889
Score ≥ 1	21 (35.6%)	12 (36.4%)	9 (34.6%)	
*HCT-ASCT applied*				
at PD during/after first-line	16 (27.1%)	7 (21.2%)	8 (30.8%)	0.840
PR after first line	2 (3.4%)	1 (3.0%)	1 (3.8%)	
First relapse	37 (62.7%)	23 (69.7%)	16 (61.5%)	
Second relapse	4 (6.8%)	2 (6.1%)	1 (3.8%)	
*Rituximab/HDMTX*				
*before induction*				
Yes	16 (27.1%)	15 (45.5%)	1 (3.8%)	<0.001
No	43 (72.9%)	18 (54.5%)	25 (96.2%)	
*Remission before*				
*HCT-ASCT*				
CR/CRu	11 (18.6%)	7 (21.2%)	4 (15.4%)	0.695
PR	13 (22.0%)	6 (18.2%)	7 (26.9%)	
SD	2 (3.4%)	1 (3.0%)	1 (3.8%)	
PD	32 (54.2%)	19 (57.6%)	13 (50.0%)	
death before control cMRI	1 (1.7%)	0	1 (3.8%)	
*Relapse/progression occurred in*				
2005–2009	9 (15.3%)	4 (12.1%)	5 (19.2%)	0.035
2010–2015	27 (45.8%)	20 (60.6%)	7 (26.9%)	
2016–2021	23 (39.0%)	9 (27.3%)	14 (53.8%)	

^a^ *p*-values for the comparison of frequencies were calculated using chi-square-tests.

**Table 2:** Multivariate analysis of prognostic factors for progression free survival and overall survival (*n* = 50 patients who completed HCT-ASCT).

Variables for PFS	HR	95% CI	*p*-Value
Age at relapse (continuous)	1.007	0.974–1.041	0.701
Relapse vs. refractory	0.850	0.380–1.901	0.692
CR/PR vs. SD/PD after induction	0.411	0.172–0.979	**0.045**
KPS <80 vs. ≥80	1.536	0.682–3.458	0.300
No involvement of DBS vs. involvement of DBS	1.430	0.632–3.240	0.391
Female vs. male	2.046	0.940–4.453	0.071
Charlson comorbidity score 0 vs. ≥1	1.484	0.672–3.276	0.328
**Variables for OS**			
Age at relapse (continuous)	1.020	0.984–1.058	0.279
Relapse vs. refractory	0.800	0.336–1.902	0.613
CR/PR vs. SD/PD after induction	0.426	0.169–1.076	0.071
KPS <80 vs. ≥80	2.406	1.026–5.643	**0.043**
No involvement of DBS vs. involvement of DBS	1.244	0.520–2.978	0.623
Female vs. male	2.426	1.046–5.627	**0.039**
Charlson comorbidity score 0 vs. ≥1	1.451	0.631–3.336	0.381

Abbreviations: CR—complete remission, DBS—deep brain structures, HR—hazard ratio, KPS—Karnofsky performance score, OS—overall survival, PD—progressive disease, PFS—progression free survival, PR—partial remission, SD—stable disease.

**Table 3:** CTC non-hematological Grade 3–4 toxicity in 50 patients who completed HCT-ASCT.

	Grade 3/4 (All Patients; *n* = 50)	Grade 3/4 (<65 Years, *n* = 30)	Grade 3/4 (≥65 Years, *n* = 20)
Infection	36 (72.0%)/4 (8.0%)	23 (76.7%)/1 (3.3%)	13 (65.0%)/3 (15.0%)
Oral Mucositis	10 (20.0%)/3 (6.0%)	7 (23.3%)/2 (6.7%)	3 (15.0%)/1 (5%)
Diarrhea	10 (20.0%)/-	5 (16.7%)/-	5 (25.0%)/-
Nausea	3 (6.0%)/-	1 (3.3%)/-	2 (10.0%)/-
Thrombosis (jugular vein)	2 (4.0%)/-	1 (3.3%)/-	1 (5.0%)/-
Increase of transaminases	-/1 (2.0%)	-/1 (3.3%)	-
Renal toxicity	1 (2.0%)/-	-	1 (5.0%)/-
Tachyarrhythmia	1 (2.0%)/-	1 (3.3%)/-	-
Hypokalemia	1 (2.0%)/-	-	1 (5.0%)/-
Delirium	1 (2.0%)/-	1 (3.3%)/-	-
Seizures	1 (2.0%)/-	-	1 (5.0%)/-
Dyspnea	1 (2.0%)/-	1 (3.3%)/-	-
Subdural hematoma during thrombocytopenia	-/1 (2.0%)	-	-/1 (5.0%)
Myelodysplastic syndrome with secondary ALL	-/1 (2.0%)	-	-/1 (5.0%)

Abbreviations: MDS-myelodysplastic syndrome, ALL-acute lymphoblastic leukemia.

## Data Availability

The data that support the findings of this study are available from the corresponding author upon reasonable request.

## Supplementary Materials

The following supporting information can be downloaded at: https://www.mdpi.com/article/10.3390/cancers14092100/s1. Supplementary Figure S1. Chemotherapy protocol (induction treatment and HCT-ASCT). Supplementary Table S1. First line treatment in *n* = 52 patients (modified Bonn protocol) [16,36]. Supplementary Table S2. Treatment and survival at relapse after HCT-ASCT (*n* = 21).
