# Supplementary material for: High-Dose Chemotherapy with Autologous Hematopoietic Stem Cell Transplantation in Relapsed or Refractory Primary CNS Lymphoma: A Retrospective Monocentric Analysis of Long-Term Outcome, Prognostic Factors, and Toxicity
Source: Cancers (Basel). 2022 Apr 23;14(9):2100. doi: 10.3390/cancers14092100 (PMC9106040; doi:10.3390/cancers14092100)
Supplement: Supplementary file 1 [file cancers-14-02100-s001.zip › cancers-1686604-supplementary.pdf]

High-Dose Chemotherapy with Autologous Hematopoietic Stem Cell Transplantation in Relapsed or Refractory Primary CNS Lymphoma: A Retrospective Monocentric Analysis of Long-Term Outcome, Prognostic Factors, and Toxicity

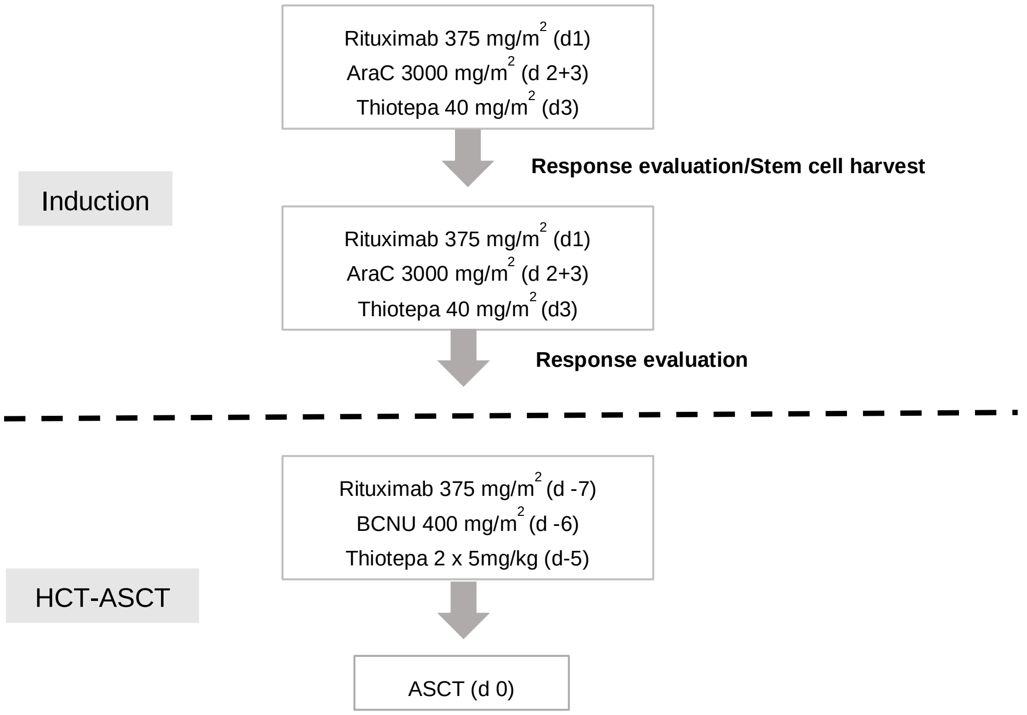

Figure S1. Chemotherapy protocol (induction treatment and HCT-ASCT). [a]one patient had received a different (busulfan-based) protocol in 2005 and one patient had been treated without rituximab in 2006. Abbreviations: AraC=cytarabine, BCNU=carmustin, HCT-ASCT=high-dose chemotherapy with autologous stem cell transplantation.

Table S1. First line treatment in n=50 patients (modified Bonn protocol, published before in [1,2][e]).

	day 0	day 1	day 2	day 3	day 4	day 5	day 6
Cycle 1-3 (1cycle = 2 weeks							
Rituximab 375-500 mg/m² IV [a]	x						
MTX 3000-5000 mg/m² IV		x					
Ifosfamide 800 mg/m² IV			x	x	x	x	
Liposomal AraC 50 mg ITH [b]			x				
Cycle 4 + 6 (1cycle = 3 weeks)							
Cytarabine 3000 mg/m² IV		x	x				
Liposomal AraC 50 mg ITH [b]			x				
MTX 2,5 mg + prednisolone 3 mg ICV [c]				x	x	x	
AraC 10 mg ICV [c]							x
Cycle 5 (1cycle = 2 weeks) [d]							
MTX 3000-5000 mg/m² IV		x					
Ifosfamide 800 mg/m² IV			x	x	x	x	
Liposomal AraC 50 mg ITH [b]			x				
MTX 2,5 mg + prednisolone 3 mg ICV [c]			x	x	x		
AraC 10 mg ICV [c]						x	

[a] patients treated 2009-2021
[b] patients treated 2005-2008
[c] patients <65 years 2010-2022, patients ≥65 years 2015-2022
[d] cycle 5 was repeated for patients < 65 years
[e] published before in [1,2])
Abbreviations: AraC, cytarabine; ICV, intracerebroventricular; ITH, intrathecal; IV, intravenous, MTX, methotrexate

References (Supplement):

1. Seidel, S.; Korfel, A.; Kowalski, T.; Margold, M.; Ismail, F.; Schroers, R.; Baraniskin, A.; Pels, H.; Martus, P.; Schlegel, U. HDMTX-Based Induction Therapy Followed by Consolidation with Conventional Systemic Chemotherapy and Intraventricular Therapy (Modified Bonn Protocol) in Primary CNS Lymphoma: A Monocentric Retrospective Analysis. *Neurological Research and Practice* **2019**, *1*, 17, doi:10.1186/s42466-019-0024-2.
2. Seidel, S.; Margold, M.; Kowalski, T.; Baraniskin, A.; Schroers, R.; Korfel, A.; Thiel, E.; Weller, M.; Martus, P.; Schlegel, U. Patients with Primary Central Nervous System Lymphoma Not Eligible for Clinical Trials: Prognostic Factors, Treatment and Outcome. *Cancers* 2021, *13*,2934.

Table S2. Treatment and survival at relapse after HCT-ASCT (n=21).

No.	Age at HCT-ASCT/ gender	Treatment at 1st relapse after HCT-ASCT	Treatment at 2nd relapse after HCT-ASCT	Treatment at 3rd relapse after HCT-ASCT	PFS after HCT-ASCT (months)	OS after HCT-ASCT (months)
1	73/m	TMZ	-	-	3	6
2	65/m	palliative	-	-	4	5
3	66/f[b]	WBRT	-	-	1	9
4	76/m	WBRT	topotecan	palliative	15	20
5	68/f	R-MTX-TMZ	MTX-ibrutinib	Focal RT	8	27
6	65/m	R-MTX-TMZ + WBRT[a]	-	-	16	21
7	68/m	WBRT	-	-	2	27
8	71/f	WBRT	-	-	5	10
9	55/f	R-MTX+ HCT-Allogeneic blood SCT	-	-	5	20
10	59/f	WBRT	-	-	4	70
11	56/f[b]	WBRT	-	-	1	4
12	57/f	Rituximab+WBRT	palliative	-	14	31
13	55/m	WBRT	-	-	5	8
14	45/m[b]	WBRT	Not known	-	2	131
15	54/f	WBRT	-	-	3	12
16	48/f	Second course HCT-ASCT	-	-	6	11
17	42/f[b]	WBRT	-	-	1	2
18	45/f	Second course HCT-ASCT	-	-	3	6
19	61/f	R-CHOP at systemic relapse	-	-	11	14
20	41/f[b]	palliative	-	-	1	2
21	67/f	palliative	-	-	3	6

[a]treatment with rituximab, methotrexate and temozolmide was stopped prematurely due to prolonged myelosuppression

[b]PCNSL refractory to HCT-ASCT

Abbreviations: HCT-ASCT=high dose chemotehrapy with autologous stem cell transplantation, OS=overall survival, PFS=progression free survival, R-CHOP=rituximab-cyclophosphamid,doxorubicin, vincristin, prednison, R-MTX-TMZ=rituximab-methotrexate-temozolomide, RT=radiotherapy, SCT=stem cell transplantation, TMZ=temozolomide, WBRT=whole brain radiotherapy
